# Screening for alcohol consumption for a worker health intervention

**DOI:** 10.1186/1940-0640-10-S2-P5

**Published:** 2015-09-24

**Authors:** Riany Brites, Ângela Maria Mendes de Abreu

**Affiliations:** 1 Universidade Federal do Rio de Janeiro Rio de Janeiro, Brazil; 2 Enfermagem de Saúde Pública Universidade Federal do Rio de Janeiro Rio de Janeiro, Brazil

## Background

The World Health Organization has been pointing out that alcohol consumption is one of the serious public health problems at present, ranking third among leading health risk factors in the world. The consumption pattern of heavy and sporadic alcohol reaches 11.5% of alcohol consumers, accounting for serious health problems. In Brazil, the pattern of alcohol use has shown alarming rates in general on average are consumed six liters of pure alcohol per capita per year. So many workers abusively consume alcoholic beverages, due to lack of knowledge of its pattern of alcohol consumption and its consequences. In this context early detection of the pattern of alcohol consumption among workers, requires further investigation in order to enable better strategies for prevention and health promotion.

The objectives are to identify the pattern of alcohol consumption and to analyze the association between social and occupational profile of workers.

## Material and methods

A descriptive study with 322 subjects who responded to the AUDIT (Alcohol Use Disorders Identification Test) and questions relating to the variables s social and occupational. Data were processed and analyzed using Epi-Info.

## Results

It was observed that 87.3% were consumption of low risk and 12.7% made use of risk, harmful and likely dependency. The episodic heavy drinking was 32.5% and 5.3% have caused problems to themselves or others. Most do not consumed alcohol in the last 12 months, but those who consumed did in quantity and high frequency.

## Conclusions

There was high prevalence of hazardous drinking, harmful and likely dependency associated with male workers and the low level of education. The sporadic heavy drinking was one of the information which alerted to the problem of alcohol consumption.
